# Evaluation of the current trend of nalidixic acid susceptibility in typhoidal Salmonellae; a marker of therapeutic failure for the fluoroquinolones

**Published:** 2011-06

**Authors:** S Abbasi, A Imtiaz, J Usman, F Kaleem, A Hassan

**Affiliations:** National University of Sciences and Technology, Rawalpindi, Pakistan

**Keywords:** Nalidixic acid resistance, Therapeutic failure, typhoid, typhoidal *Salmonella*

## Abstract

**Background and Objectives:**

Typhoid is a major health problem faced by the developing countries like Pakistan. More than 20 million cases are reported annually worldwide. Currently fluoroquinolones are the drugs of choice to treat typhoid fever. *In vivo* resistance to fluoroquinolones leading to therapeutic failure is developing rapidly and is becoming a major concern for the clinicians. The objective of this study was to determine the sensitivity pattern of Nalidixic acid over the last four years

**Material and Methods:**

A descriptive cross sectional study was carried out at the Microbiology Department of the Army Medical College, National University of Sciences and Technology, Rawalpindi from January 2006 to December 2009. All the isolates were dealt with standard microbiological procedures. The antimicrobial sensitivity of Nalidixic acid and Ciprofloxacin was determined using Kirby-Bauer disc diffusion method as per the guidelines of Clinical and Laboratory Standard Institute (CLSI).

**Results:**

Out of 240 isolates, 111 were *Salmonella typhi* and 129 were *Salmonella paratyphi* A. The resistance of the typhoidal *Salmonella* to Nalidixic acid has reached significant levels and it seems only a matter of time when hundred percent resistance will be encountered. All isolates were sensitive to Ciprofloxacin on disc diffusion method.

**Conclusion:**

Resistance to Nalidixic acid predicting therapeutic failure with fluoroquinolones is on a steady rise.

## INTRODUCTION

Typhoid has been a menace to humanity for centuries. It caused the fall of Athens in 430 B.C., ending the Golden Age of Pericles (1). It continues to plague the developing world even in the 21st century. The morbidity of typhoid fever is highest in Asia with 93% of global episodes occurring in this region. Southeast Asia has an estimated incidence of 110 cases/100,000 population, which is the third highest incidence rate for any region (2).

In Asia, Pakistan has the highest incidence of *Salmonella typhi* (*S. Typhi)* and *Salmonella paratyphi A* (*S. Paratyphi* A) (3). In the 1970's, antibiotic resistance against conventional antityphoid drugs emerged for the first time. By the mid 1990's multidrug resistant strains, i.e. those having simultaneous resistance to the three conventional antityphoid drugs (chloramphenicol, trimethoprim-sulfamethoxazole and ampicillin), became rampant all over the world. Fluoroquinolones then became the treatment of choice for typhoid fever (4). Widespread, injudicious use of these drugs, has led to the emergence of resistance.

So far, *in-vitro* fluoroquinolone resistance has not been reported on a widespread scale in Pakistan. Therapeutic failure with fluoroquinolones, however, is being reported from various parts of Pakistan with increasing frequency (5). In the laboratory, using the routine disc diffusion tests, isolates with decreased *in vivo* susceptibility to ciprofloxacin appear susceptible (6).

Nalidixic acid is a naphthyridone but is considered to be the first of the synthetic quinolone antibiotics. It has structural similarity with the newer generation quinolones. A single point mutation in the quinolone resistance-determining region (QRDR) of the gene *gyrA* in Salmonella leads to resistance against nalidixic acid as well as decreased *in vivo* ciprofloxacin susceptibility (7). Thus, resistance to nalidixic acid is used as an indicator of decreased susceptibility to the fluoroquinolones (8). The aim of this study was to determine the current trend in the nalidixic acid sensitivity in *Typhoidal Salmonellae*.

## MATERIALS AND METHODS

A descriptive cross sectional study carried out at the Microbiology Department, Army Medical College, National University of Sciences and Technology (NUST), Rawalpindi, from January 2006 to December 2009.

Blood culture samples were received from the wards of an 1,100 bed tertiary care hospital, the Military Hospital (MH) of Rawalpindi. The samples were collected aseptically using a disposable syringe. The top of the culture bottle was cleaned with iodine immediately before addition of blood. Five ml of venous blood drawn from adults was added to fifty ml of sterile Brain Heart Infusion (BHI) (Merck). For children, the ratio used was 3 ml of blood in 30 ml BHI (Merck).

Subcultures were made on Blood and MacConkey's agar (Oxoid, Basingstoke, UK) at 24 hr, 48 hr, and 5^th^ and 7^th^ day. Cultures that showed no growth till the seventh day were reported as negative. The species were identified on the basis of morphology and standard biochemical tests using API 20E (Biomerieux, France) and the serovars were confirmed as *S. Typhi* and *S. Paratyphi* A by serological tests using standard anti-sera (Bio-Rad) using polyvalent A-S, O9 for *S.Typhi* and O2 for *S. Paratyphi* A.

Antimicrobial susceptibility was tested for nalidixic acid and ciprofloxacin using discs of 30 µg and 5 µg respectively (Britania, Argentina) on Mueller-Hinton agar (Britania) by Kirby-Bauer disc diffusion method as per the Clinical and Laboratory Standards Institute (CLSI) guidelines (9). The plates were incubated at 37°C for 24 hours. The control strain used was *Escherichia coli* ATCC 25922. The data was analyzed using SPSS version 17.0.

## RESULTS

**Fig. 1:**
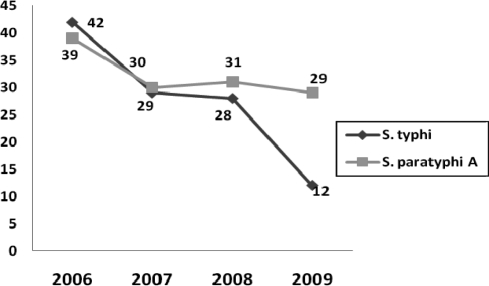
Number of isolates per year.

A total of 240 typhoidal *Salmonellae* were isolated during the study period. Out of these, 111 were *S*. *Typhi* and 129 were of *S*. *Paratyphi A*. There was an increase in the isolation rate of *S. Paratyphi* A compared to *S. Typhi* in 2007-2009. (Fig. 1) The subjects age ranged from one to ninety years old (mean = 33.7 years). The male to female ratio was 17.1:1.

All 240 isolates were sensitive *in-vitro* to ciprofloxa-cin throughout these four years. The sensitivity was checked using 5 µg Ciprofloxacin discs (Britania, Argentina) on Mueller–Hinton agar (Britania) by Kirby-Bauer disc diffusion method.

**Fig. 2:**
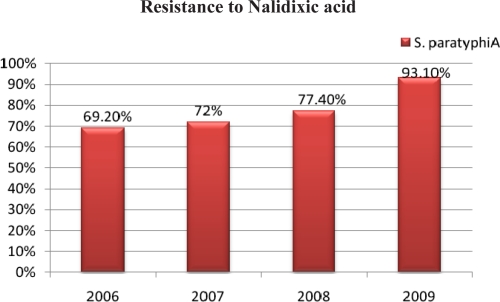
Nalidixic acid resistance in *S. paratyphi A*.

The resistance to nalidixic acid in *S*. *Paratyphi* A increased with each subsequent year. In 2006, it was 69%. It increased slightly to 72% in 2007, became 77% in 2008 and in 2009 more than 93% of the isolates of *S*. *Paratyphi A* were resistant to nalidixic acid. (Fig. 2)

**Fig. 3:**
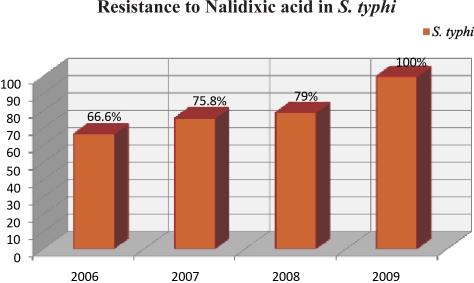
Nalidixic acid resistance in *S*. *typhi*.

A similar pattern was observed in *S*. *Typhi*, with 66% of isolates being resistant to nalidixic acid in 2006. This increased to 75 and 79% in 2007 and 2008 respectively. In 2009, all the isolates were resistant to nalidixic acid. (Fig. 3)

**Fig. 4:**
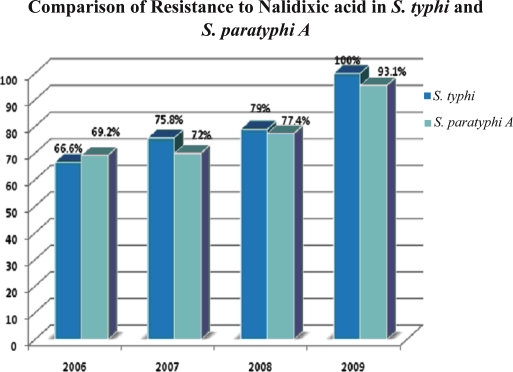
Nalidixic acid resistance in *S. typhi* and *S. paratyphi A*.

A comparison of the resistance pattern of both serovars revealed that the resistance to nalidixic acid is rising equally in both. (Fig. 4)

## DISCUSSION

Typhoid continues to be a major health problem in Pakistan. Its treatment still remains a challenge due to the rapid emergence of antibiotic resistance in the causative strains. In an attempt to determine the current resistance pattern of the *typhoidal Salmonellae* in our region, we compared the sensitivity trends of *Salmonella enterica* subspecies *typhi* and *paratyphi A* isolated between 2006 and 2009.

In our study, several interesting trends were observ-ed. A decrease in the isolation rate of *Salmonella* over the past 4 years was observed (from 81 in 2006 to 41 in 2009). This concurs with the study conducted at the Armed Forces Institute of Pathology in which the isolation rate of *typhoidal Salmonellae* decreased significantly from 147 in 1996 to 49 in 2003 (10). This however, doesn't suggest that the incidence of typhoid fever has gone down, as the various factors responsible for the endemicity of typhoid in Pakistan have not changed.

Another trend seen was the increase in the relative proportion of *S*. *Paratyphi A* isolates compared to *S*. *Typhi* (53.04% *S*. *Paratyphi A* and 47.39% *S*. *Typh*i). The incidence of enteric fever caused by *S. Paratyphi A* has been increasing. This is similar to the reports from a regional study by Thankhiwale et al. (11).

In a study conducted in the United States by Michael F Lynch et al., 38 percent resistance to nalidixic acid was reported in isolates from the year 1999 to 2006 (12). In India, *S. Paratyphi A* isolation rates in enteric fever cases were reported to be 26, 27 and 52% in 2003, 2004, and 2005 respectively (13). Chandel et al**. attribute this dramatic increase in incidence of enteric fever by *S. Paratyphi A* to the widespread use of vaccines and quinolones against *S. Typhi* in the past decade (14). In our study, the incidence of *S. Paratyphi A* isolates was found to be 48, 49, 53 and 71% in 2006, 2007, 2008 and 2009 respectively.

Nalidixic acid screening has been used to detect and predict decreased fluoroquinolone susceptibility in typhoidal *Salmonella*. Identification of nalidixic acid resistance by the disk diffusion method provides a sensitivity of 100% and a specificity of 87.3% (8).

Nalidixic acid resistance was found to be increasing in both *S. Typhi* and *S. Paratyphi A* during the last four years. All *S. Typhi* have developed resistance to nalidixic acid whereas *S. Paratyphi A* also seems to be catching up fast. This is in sharp contrast to the study carried out in 2001-2002 in the same setting, in which only 4% of the isolates were found to be resistant to nalidixic acid (15).

Despite the increasing resistance to nalidixic acid, all 240 isolates were found to be sensitive to ciprofloxacin *in-vitro*. This is in accordance with a regional study by Asna, et al**. in 2003 that found that the isolates with decreased *in vivo* susceptibility to ciprofloxacin appear susceptible with routine disc diffusion tests (6).

The first case of fluoroquinolone treatment failure in typhoid Salmonellae was reported in Pakistan in 1993 (16). Since then the incidence of such cases has been increasing. Inability to identify reduced susceptibility to fluoroquinolones by the standard disk diffusion techniques further compounds the problem. A reappraisal and revision of the current guidelines for detection of this resistance is necessary.

In conclusion, there is a definite shift in the distribution as well as antimicrobial susceptibility of typhoidal Salmonellae in Pakistan. The resistance to nalidixic acid is on a steady rise in both serovars. This foretells a rise in therapeutic failure with the fluoroquinolones. It is imperative that these drugs be used judiciously to limit the spread of resistance and new, reliable treatment options must be sought before untreatable typhoid fever becomes a major problem.

## References

[1] Papagrigorakis MJ, Yapijakis C, Synodinos PN, Baziotopoulou-Valavani E (2006). DNA examination of ancient dental pulp incriminates typhoid fever as a probable cause of the Plague of Athens. Int J Infect Dis.

[2] Crump JA, Luby SP, Mintz ED (2004). The global burden of typhoid fever. Bull WHO.

[3] Ochiai RL, Wang XY, von Seidlein L, Yang J, Bhutta ZA, Bhattacharya SK (2005). *Salmonella Paratyphi* A rates in Asia. Emerg Infect Dis.

[4] Parry CM, Hien TT, Dougan G, White NJ, Farrar JJ (2002). Typhoid fever. N Engl J Med.

[5] Ahmad R, Mahmood A, Zaidi S (2005). Ciprofloxacin treatment failure in typhoid fever case, Pakistan. East Mediterr Health J.

[6] Asna SM, Haq JA, Rahman M (2003). Nalidixic acid-resistant *Salmonellaenterica* serovar *typhi* with decreased susceptibility to ciprofloxacin caused treatment failure: a report from Bangladesh. Jpn J Infect Dis.

[7] Ouabdesselam S, Tankovic J, Soussy CJ (1996). Quinolone resistance mutations in the *gyrA* gene of clinical isolates of Salmonella. Microb Drug Resist.

[8] Hakanen A, Kotilainen P, Jalava J, Sutonen A, Huovinen P (1999 November). Detection of decrease fluroquinolone susceptibility in Salmonellas and validation of nalidixic acid screening test. J Clin Microbiol.

[9] Clinical and Laboratory Standards Institute (2009). Performance standards for antimicrobial susceptibility testing, 19th informational supplement.

[10] Butt T, Ahmad RN, Salman M, Kazmi SY (2005). Changing trends in drug resistance among typhoid Salmonellae in Rawalpindi, Pakistan. East Mediterr Health J.

[11] Tankhilwala SS, Agarwal G, Jalgaonkar SV (2003). An unusually high occurrence of *Salmonella enterica* serotype *paratyphi A* in patients with enteric fever. Ind J Med Res.

[12] Lynch MF, Blanton EM, Bulens S, Polyak C, Vojdani J, Stevenson J (2009). Typhoid fever in the United States, 1999–2006. JAMA.

[13] Aggarwal A, Singh A, Oberoi A (2007). A three-year retrospective study on the prevalence, drug susceptibility pattern and phage types of *Salmonella enteric* subspecies *typhi* and *paratyphi* in Christian Medical College and Hospital, Ludhiana, Punjab. J Indian Academy C Med.

[14] Chandel DS, Chaudhry R, Dhawan B, Pandey A, Dey AB (2000). Drug-resistant Salmonella enterica Serotype *Paratyphi A* in India. Emer Infec Dis.

[15] Hannan A, Butt T, Islam SN (1993). First quinolone resistant typhoidal Salmonella. Pak Armed F Med J.

